# Cross-sectional study of *Staphyloccus lugdunensis* prevalence in cats

**DOI:** 10.1038/s41598-020-72395-8

**Published:** 2020-09-22

**Authors:** Karolina Bierowiec

**Affiliations:** Division of Infectious Diseases and Veterinary Administration, Department of Epizootiology and Clinic of Birds and Exotic Animals, Faculty of Veterinary Medicine, Wroclaw University of Environmental and Life Sciences, Pl. Grunwaldzki 45, 50-366 Wroclaw, Poland

**Keywords:** Microbiology, Bacteriology, Infectious diseases, Bacterial infection

## Abstract

*Staphylococcus lugdunensis* is a commensal bacterium in humans and other animals that can cause serious infections. The aim of this research was to estimate the frequency of *S. lugdunensis* in pet cats and to characterize the *S. lugdunensis* isolates obtained. The prevalence of *S. lugdunensis* was 0.77% (4/523) in healthy cats and 1.23% (1/81) in sick cats. The isolates (N = 5), which colonized conjunctival sacs, nares, and the anus, were almost fully phenotypically sensitive to antibiotics, but harbored resistance genes to four chemotherapeutic groups. Their sequence types (STs) included ST2, ST3, ST9, and ST15. There was detected a far lower prevalence of *S. lugdunensis* in pet cats than is reported in the human population. Nevertheless, the phenotypic and genotypic properties of *S. lugdunensis* isolates found in the current study were very similar to those described previously in isolates of human origin.

## Introduction

Coagulase-negative staphylococci (CoNS) are common commensal bacteria of the skin and mucosal membranes of mammals^1,2^. The importance of CoNS as pathogens that cause severe hospital and community acquired infections in both human and veterinary patients has gained attention in recent years. Notably, the pathogenicity and virulence characteristics of the CoNS species *Staphylococcus lugdunensis* have been described as comparable to those of *Staphylococcus aureus.* In healthy humans, *S. lugdunensis* colonization of the skin (especially of the groin, toes, and axillae) has been reported to be three-fold more frequent than that of *S. aureus*, which colonizes mainly the nose^1^. However, clinically significant *S. lugdunensis* infections in humans, including infections of the skin, pelvic soft tissues, and lower extremities (including the feet) have been reported, as have cases of infective endocarditis, bone and joint infections, and septicemia due to *S. lugdunensis* infection^1,3–6^. Though *S. lugdunensis* has been isolated from healthy dogs and cats, severe *S. lugdunensis* infections of the urogenital tract, respiratory tract, deep tissues, and wounds have also been reported in dogs and cats^2,7^.

Although *S. lugdunensis* is gaining attention as a cause of severe human infection, especially in cardiology and orthopedy, there is little recognition of the bacterium in veterinary medicine^4,5^. *S. lugdunensis* colonization of pets could potentially be dangerous for people. Therefore, the aims of the present study were to describe the prevalence of *S. lugdunensis* in a cat population sample, to characterize the virulence potential of *S. lugdunensis* isolates from cats, and to evaluate factors that may predispose cats to *S. lugdunensis* colonization or infection. Present work follows up from a previous study focusing on the prevalence of staphylococci in cats^8^.

## Materials and methods

Isolates were collected from cats that had been screened for *Staphylococcus *spp. colonization during the period of 2013–2019 in the Department of Epizootiology and Clinic for Birds and Exotic Animals at Wrocław University of Environmental and Life Sciences in Poland. Specimens were collected from two groups: healthy cats; and cats with symptoms of a bacterial infection of the upper respiratory tract, skin, or wound^8^. The research project was submitted to the Local Ethics Committee for Animal Experiments in Wrocław, Hirszfeld Institute of Immunology and Experimental Therapy, Polish Academy of Sciences. Due to the noninvasive samples collection procedure, the Ethics Committee qualified the study as research which therefore did not require any further approval from the Ethics Committee. All methods described were approved by Wroclaw University of Environmental and Life Sciences and performed in compliance with the relevant guidelines and regulations for good laboratory practice. Each cat owner has informed consented to take part in this study and filled out the proper documentation. Swabs were used to collect samples from four anatomical sites (nares, conjunctival sacs, groin, anus) in 523 healthy and 81 sick cats, and additionally from a wound, if present, on sick cats. Additionally, each cat owner filled out the questionnaire considering potential factors connected with staphylococci colonization in cats under investigation, such as animal features (age, sex, breed, medical history) and household's factors (medical occupations or previous hospitalization of household members, other animals kept in the same household and their medical history).

Collected swabs were placed into 2 ml of liquid brain–heart infusion broth (BHI) (Oxoid, United Kingdom) and incubated at 37 °C for 24 h. Then the material was subcultured in Mannitol Salt Agar and blood agar plate (Oxoid, United Kingdom) and incubated for 24 h. The preliminary identification of staphylococci was according to the colony morphology, Gram staining, and detection of enzyme production (coagulase tube test; IBSS Biomed, Poland). *S. lugdunensis* species was identified by polymerase chain reaction (PCR) with species-specific primers performed according to previously detailed reaction conditions^9^. All the isolates of *S. lugdunensis* were screened for antibiotic susceptibility using both methods: phenotypic [disc diffusion and MIC (Sensititre, Staphylococcus MIC plates, Thermo Fisher Scientific, Waltham, MA, USA)] and genotypic (detections of genetic determinants of resistance). The phenotypic and genotypic antibiotic resistance of each isolate was determined as described previously^10^. *S. lugdunensis* was tested for slime production by the Congo red agar method, microtiter plate test, and a standard PCR technique for *ica*A and *bap* genes detection^10^. Prevalence rates of *S. lugdunensis* were calculated for the sick cat group and for the healthy cat group by the bootstrap method in the R Statistical Package v. 2.11.1. The statistical analysis of potential risk factors, associated with *S. lugdunensis* colonization in cats under investigation, was not performed due to a small number of colonized animals in both groups of cats.

*Staphylococcus lugdunensis* allele sequence types (STs) were determined by multi-locus sequence typing (MLST) with a sequence alignment tool (MEGA X 10.1.) focusing on seven housekeeping loci described by Chassain et al.^11^. MLST was performed with sequence and profile data from *Institute Pasteur* https://bigsdb.pasteur.fr/staphlugdunensis/staphlugdunensis.html). I determined the ST of each *S. lugdunensis* isolate under investigation by using the search tool with a combination of *S. lugdunensis* loci in the PasteurMLST database (https://bigsdb.pasteur.fr/cgi-bin/bigsdb/bigsdb.pl?db=pubmlst_staphlugdunensis_isolates&page=profiles).

## Results

In total, five distinct *S. lugdunensis* isolates were identified, including four from healthy cats and one from the conjunctival sack of a cat with conjunctivitis and sneezing symptoms (GenBank accession numbers of 16S RNA sequences of isolated *S. lugdunensis*, MT1880032–MT1880036). In most cases, *S. lugdunensis* was isolated alone; in the conjunctival sack of one healthy cat it was isolated with *S. epidermidis*. Information about the cats colonized with *S. lugdunensis* is summarized in Table 1.

**Table 1:** Characteristics of cats colonized with *S. lugdunensis* and isolate origin site.

Isolate ID	Health status	Breed	Sex	Age (months)	Housing	Origin site
P62	Healthy	Russian Blue	Male	8	Group of cats	Anus
N281	Healthy	Cornish Rex	Male	72	Registered cattery	Nares
O465/2	Healthy	Mixed-breed	Male	86	Group of cats	Conjunctival sacs
P1512	Healthy	Mixed-breed	Female	24	Solitary	Anus
O514	Sick	Devon Rex	Female	30	Registered cattery	Conjunctival sacs

The prevalence of *S. lugdunensis* in cats from Wrocław city area was 0.77% [95% confidence interval (CI) 0.02–1.51%] in healthy cats and 1.23% (95% CI 0.0–3.64%) in sick cats. The antibiotic resistance profiles and biofilm-forming properties of the investigated isolates are presented in Table 2. Although isolates exhibited robust biofilm formation on polystyrene plates, none harbored the *ica*A or *bap* gene. Four different *S. lugdunensis* STs were found (Table 2). The ST of *S. lugdunensis* isolates were deposed in the Institut Pasteur MLST database (https://bigsdb.pasteur.fr/staphlugdunensis/staphlugdunensis.html), identified as isolates 113–117.

**Table 2:** STs, antibiotic resistance profiles, and biofilm-forming properties of investigated *S. lugdunensis* isolates.

Isolate ID	ST	Antibiotic resistance profile	Biofilm production^a^
P62	9	SMX/*bla*Z; *tet*(K), *tet*(M), *tet*(O), *tet*(L), *erm*B, *erm*C, *van*A	Strong
N281	2	*bla*Z; *tet*(K), *tet*(M), *erm*B, *erm*C, *van*A	Strong
O465/2	2	SMX/*bla*Z; *tet*(K), *tet*(M), *erm*B, *erm*C, *van*A	Strong
P1512	3	SMX/*bla*Z; *tet*(K), *tet*(M), *erm*B, *erm*C, *van*A	Strong
O514	15	AMP; SMX/*bla*Z; *tet*(K), *tet*(M), *erm*B, *erm*C, *van*A	Strong

*SMX* sulfamethoxazole, *AMP* ampicillin, *blaZ* penicillin resistance gene, *tet(K)(M)(O)(L)* tetracycline resistance genes, *ermB/C* macrolide-lincosamide-streptogramins resistance genes, *vanA* glycopeptide resistance gene.

^a^According to microtiter plate assay results; weak biofilm formers, 0.265 ≤ A570 < 0.422; medium-positive biofilm formers, 0.422 ≤ A570 < 0.844; strong biofilm formers, A570 ≥ 0.844.

## Discussion

The present data indicate that *S. lugdunensis* is likely to be much more rare among pet cats population under investigation (~ 1%) than among humans 30–50%^12^. Notwithstanding, given the potential risk of *Staphylococcus* interspecies transmission, especially to human surgery patients, the prevalence of *S. lungdunensis* in pets should be monitored.

This study provides some information about *S. lugdunesnsis* characteristics and carriage sites in cats, but, despite sampling a representative group of cats, the small number of isolates found limits the power of the analysis. There was observed colonization of the perineum, as has also been documented in humans^1,13^. Interestingly, two isolates were found in cats’ conjunctival sac samples. To the best of knowledge, there have been no reports of conjunctivitis or keratitis caused by *S. lugdunensis* in pets. However, there have been a few such cases in human patients^14,15^. Moreover, the identification of *S. lugdunensis* as a potential causative pathogen of cat bacterial conjunctivitis may be indicative of a wide spectrum of possible infection sites for the bacterium, which is relevant to both veterinary and human medicine.

Contrary to other CoNS, *S. lugdunensis* remains sensitive to most antibiotics despite its pathogenicity^1,4–6,12^. Among the presently analyzed isolates, only resistances to sulfamethoxazole and ampicillin were identified. Others have identified penicillin- and erythromycin-resistant *S. lugdunensis* isolates, as well as *S. lugdunensis* isolates with susceptibility to all antibiotics tested^3,12^. Reports of *S. lugdunensis* isolates collected from humans harboring antibiotic resistance genes, especially genes that can confer resistance to penicillin (*bla*Z), macrolides (*erm*B/C), and tetracyclines (*tet*K/L/M/O)^16^ indicate that *S. lugdunensis* has the potential to develop phenotypic resistance to antibiotic drugs. Furthermore, a report showing that bacteria exhibit lower antibiotic resistance when they are grown in plankton than when they are grown in a biofilm, indicate that standard in vitro phenotypic antibiotic resistance testing may not fully reflect the in vivo efficiency of chemoterapeutics towards *S. lugdunensis*^17^. The present observation of strong *S. lugdunensi*s biofilm-forming properties is consistent with prior observations^18,19^

There are currently 20 *S. lugdunensis* STs catalogued in the Institut Pasteur MLST database^11^. The STs identified in the current study, ST2 and ST3, are the most frequent *S. lugdunensis* STs found in humans, accounting for 30% of deposed isolates thus far. Further research into the occurrence of *S. lugdunensis* in pet animals is needed to elucidate the pathogenic potential of this ubiquitous species and its interspecies transmission risk.

## Conclusion

The current study characterized the possible carriage sites for *S. lugdunensis* in cats in Wrocław city area, which could be used in future research design. There was detected a far lower prevalence of *S. lugdunensis* in pet cats than is reported in the human population. Nevertheless, the phenotypic and genotypic properties of *S. lugdunensis* isolates found in the current study were very similar to those described previously in isolates of human origin. Further studies are necessary, to better understand the emergence of as a veterinary and zoonotic pathogen, to evaluate the risks of interspecies transmission and potential factors connected with *S. lugdunensis* colonization, and to determine appropriate household infection control practices.

## Data Availability

All data are presented in the main paper.
